# The Effect of Angiotensin II Receptor Blockers in Patients with Hypertrophic Cardiomyopathy: An Updated Systematic Review and Meta-analysis of Randomized Controlled Trials

**DOI:** 10.31083/j.rcm2304141

**Published:** 2022-04-12

**Authors:** Basel Abdelazeem, Kirellos Said Abbas, Soban Ahmad, Hasan Raslan, Fatma Labieb, Pramod Savarapu

**Affiliations:** ^1^Internal Medicine, McLaren Health Care, Flint, MI 48532, USA; ^2^Internal Medicine, Michigan State University, East Lansing, MI 48823, USA; ^3^Faculty of Medicine, Alexandria University, 21131 Alexandria, Egypt; ^4^Internal Medicine, East Carolina University, Greenville, NC 27858, USA; ^5^Faculty of Medicine, Aleppo University, 15310 Aleppo, Syria; ^6^Faculty of Medicine, Beni Suef University, 62521 Beni Suef, Egypt; ^7^Internal Medicine, Ochsner Louisiana State University Health, Monroe, LA 71202, USA

**Keywords:** hypertrophic cardiomyopathy, angiotensin II receptor blockers, left ventricular mass, systolic blood pressure, systematic review, meta-analysis

## Abstract

**Background:**

Angiotensin receptor blocker (ARB) therapy has been evaluated to slow down the disease progression in patients with hypertrophic cardiomyopathy (HCM), but there is scarce evidence available to date. Therefore, our meta-analysis aimed to explore the efficacy of ARB therapy as a potential disease-modifying treatment in patients with HCM.

**Methods:**

A literature search was performed using PubMed, Scopus, Web of Science, Embase, Cochrane library, and Clinicaltrials.gov databases from inception to December 13th, 2021. We included only randomized controlled trials (RCTs). The quality of included studies was assessed by the Cochrane Collaboration’s tool. Primary outcomes included the reduction in left ventricular mass and improvement in other echocardiographic features of myocardial dysfunction. The secondary outcome was a net reduction in systolic blood pressure. Meta-analysis was performed using pooled standardized mean difference (SMD) and corresponding 95% confidence interval (CI).

**Results:**

A total of 1286 articles were screened. Seven RCTs met the inclusion criteria representing a total of 397 patients with HCM (195 patients were in the ARB group). ARB treatment was associated with significant reduction in left ventricular mass (SMD: –0.77; 95% CI: –1.40, –0.03; *p* = 0.04). ARB therapy was also associated with a significant reduction in systolic blood pressure (SMD: –0.33; 95% CI: –0.61, –0.05: *p* = 0.02).

**Conclusions:**

ARB therapy is associated with a marked reduction in left ventricular mass and systolic blood pressure in patients with hypertrophic cardiomyopathy. We recommend further studies with a larger patient population size to confirm the findings of our meta-analysis.

**Clinical Trial Registration:**

OSF Registries, DOI: 10.17605/OSF.IO/DAS7C.

## 1. Introduction 

Hypertrophic cardiomyopathy (HCM) is the most common inheritable disease of the myocardium that is caused by genetic mutations of sarcomeric myofilaments [1, 2]. HCM is a global disease with a prevalence of 1:500 in the general adult population, equally affecting both men and women [3]. HCM carries a significant risk for diastolic heart failure, ventricular arrhythmias, and sudden cardiac death (especially in competitive athletes) [4]. HCM can be clinically diagnosed with two-dimensional echocardiography showing maximal left ventricular end-diastolic (LVED) wall thickness of ≥15 mm in the absence of pressure overload in adults [5, 6]. Genetic testing and family history of HCM can be helpful in patients who do not meet echocardiographic LVED wall thickness criteria [7].

Angiotensin II triggers the production of several trophic and pro-fibrotic factors that lead to myocardial hypertrophy and interstitial fibrosis [8]. Theoretically, angiotensin II receptor blockers (ARBs) should diminish the progression of LV hypertrophy and fibrosis by decreasing levels of pro-fibrotic factors. In addition, genetic studies of the renin-angiotensin-aldosterone system systems reported that genetic polymorphisms might influence the phenotypic changes observed in HCM [8]. In the past, randomized controlled trials (RCTs) failed to report any additional benefit of ARB therapy as compared to standard medical therapy consisting of negative inotropic agents including beta-blockers and non-dihydropyridine calcium channel blockers [2, 9, 10, 11, 12]. A previously published meta-analysis by Liu *et al*. [13] comprising those RCTs also concluded no net benefits of ARBs on ventricular hypertrophy in hypertrophic cardiomyopathy.

In a recent multicenter RCT performed by Ho *et al*. [14], valsartan has shown promising results in attenuation of phenotypic expression of disease in patients with HCM. They reported that that valsartan not only attenuated the progression but also improved the prognosis as it decreased type I collagen synthesis and secondary to renin-angiotensin-aldosterone system activation, which is associated with systolic dysfunction by breaking through the aldosterone.

Given the clinical importance of this topic and in light of the newer data, we performed this updated systematic review and meta-analysis aiming to evaluate the effectiveness of ARB’s therapy in patients with HCM.

## 2. Materials and Methods

This review was carried out according to the guidelines provided in Preferred Reporting Items for Systematic Reviews and Meta-Analysis [15, 16] (**Supplementary Table 1** and **Supplementary Table 2**, **Supplementary Material**). The study protocol was registered in OSF Registries with DOI: 10.17605/OSF.IO/DAS7C.

### 2.1 Data Sources and Search Strategy

We systematically searched a range of databases (PubMed, Scopus, Web of Science, Embase, Cochrane library, and Clinicaltrials.gov) from inception to December 13th, 2021. The keywords used for searching include “angiotensin II receptor blocker”, “ARBs”, “hypertrophic cardiomyopathy”, “HCM”, and “Randomized control trials”. We provide the complete research strategies and results from the included databases in **Supplementary Table 3**, **Supplementary Material**. In addition, the reference of related articles and reviews were manually reviewed and searched to identify additional studies of relevance. Publication language is limited to English.

### 2.2 Study Selection and Eligibility Criteria

Studies are eligible to be included if the following criteria are met: (1) studies must be RCTs that included adults aged ≥18 years, (2) studies evaluated the effect of ARBs in HCM, (3) Trials with primary reports of left ventricular (LV) mass and other echocardiographic features of myocardial dysfunction. We excluded Non-randomized trials and observational studies. The search results were uploaded into the Covidence software, and all duplicates were recognized and removed. The remaining titles and abstracts were screened independently by the two authors (HR and FL). The full text of the potentially relevant studies was then retrieved and evaluated for eligibility through a full-text review. A third author (KSA) resolved any disagreements in the screening process.

### 2.3 Data Extraction

Two reviewers (HR and FL) independently extracted the following data from the included RCTs: (1) LV mass reduction, (2) systolic blood pressure, (3) Left atrial (LA) volume, (4) Left ventricular ejection fraction (LVEF), (5) LV wall thickness, (6) early diastolic velocity (Ea), (7) early to late transmitral flow velocities (E/A) ratio, and (8) LV fibrosis. Any discrepancies in data extraction between the two reviewers were judged by a third reviewer (KSA).

### 2.4 Risk of Bias Assessment 

Assessment of probable biases was done through Cochrane Collaboration’s risk of bias tool (ROB 1) [17]. ROB 1 tool assesses quality through evaluating random sequence generation, concealment in allocation, blinding, reporting, and possible other biases.

### 2.5 Outcomes of Interest

Our primary outcomes are a variety of multi-measures that represent heart function. Those are the changes in left ventricular mass, left ventricular wall thickness, left ventricular ejection fraction, and the progression of left ventricular fibrosis. In addition to early diastolic velocity, early to late (atrial) transmittal flow velocities (E/A) ratio, and left atrial volume.

Our secondary outcomes were the changes in systolic blood pressure.

### 2.6 Statistical Analysis

Pooled standardized mean difference (SMD) and corresponding 95% confidence interval (CI) were used in our meta-analysis due to heterogeneity in the methodologies of the included studies. We used the random-effects method (DerSimonian-Laird method) and considered a *p*-value less than 0.05 statistically significant for all analyses. Statistical heterogeneity was assessed with the Higgins’ and Thompson’s $I^{2}$ statistic. We considered *p *≤ 0.05 or $I^{2}$ >50% having a high level of heterogeneity. Due to the missed standard deviations (SDs) and inability to estimate it using correlation coefficient, we followed Follmann *et al*.’s [18] recommendation to impute SDs using the largest value between the included studies. Subgroup analysis and sensitivity analysis were done for the significant outcomes. Subgroup analysis was done according to the type of ARBs, and sensitivity analysis was done by omitting one study sequentially. We didn’t use the Egger test to investigate the publication bias due to the insufficient number of the included studies. Forest plots were generated using Review Manager software(version 5.4, The Nordic Cochrane Centre, The Cochrane Collaboration: Copenhagen, Denmark) [19]. All meta-analysis was performed by KSA and reviewed by BA.

## 3. Results

### 3.1 Study Identification and Selection

**Fig. 1.**
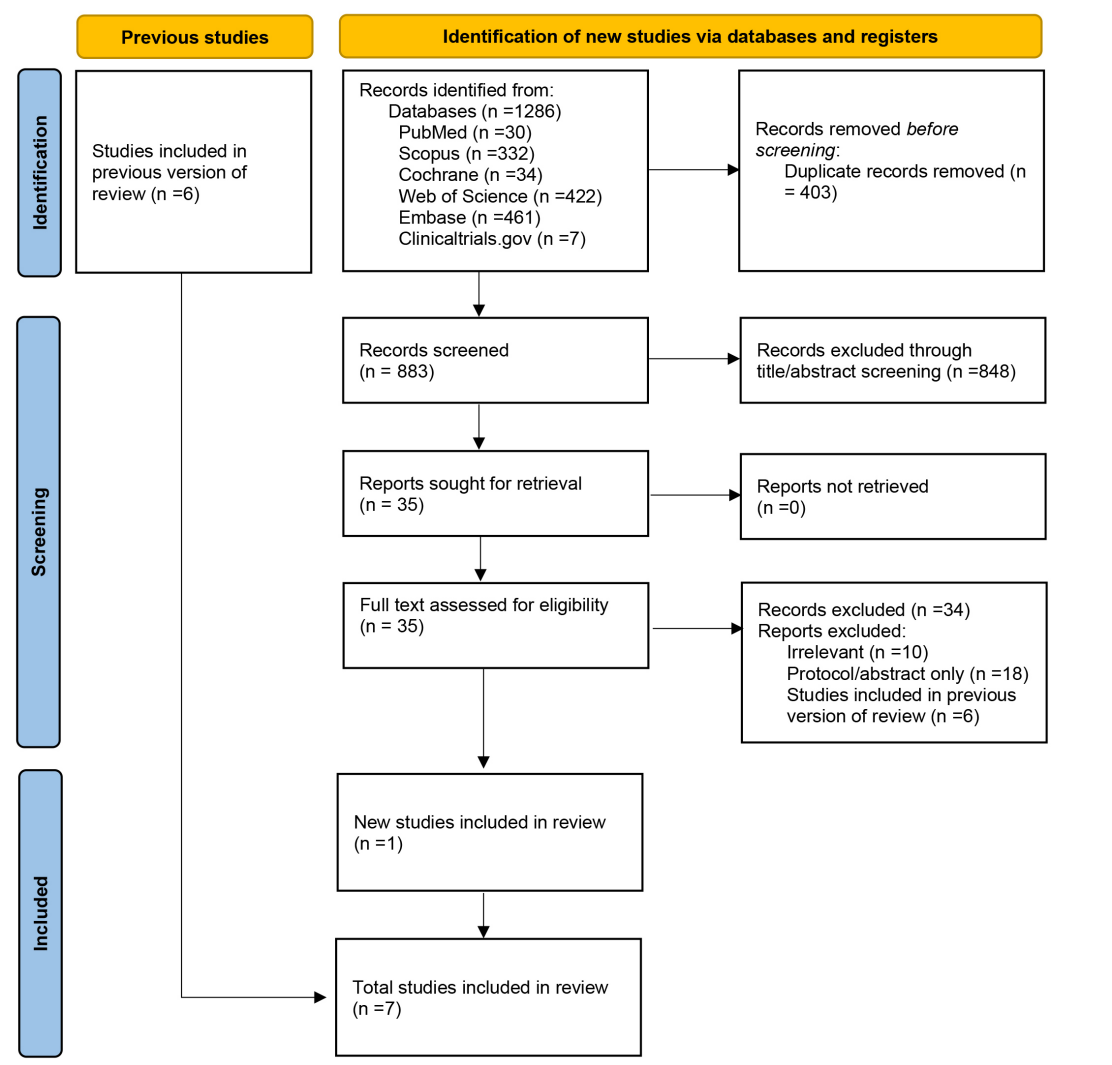
**PRISMA 2020 flow diagram for updated systematic reviews**. The PRISMA diagram included searches of databases, registers, and other sources and the various reasons for the excluded articles.

There were 1286 articles identified from our literature search, of which 403 were excluded as duplicates. A total of 883 articles underwent title, and abstract screening, then 35 were eligible for full-text evaluation. Finally, only seven RCTs met our inclusion criteria and were included in the meta-analysis [2, 9, 10, 11, 12, 14, 20]. Fig. 1 PRISMA flow diagram shows the process of selection and the various reasons for the excluded articles.

### 3.2 Characteristics of Included Studies

Table 1 (Ref. [2, 9, 10, 11, 12, 14, 20]) displays the summary of the included RCTs. The aggregate study population included a total of 397 HCM patients with 195 (49.24%) in the ARB group [2, 9, 11, 12, 14, 20] with males representing 65.40 % of the population . The ARB group had a mean age of 38.67 ± 11.82 years, and the placebo or non-ARB group had a mean age of 39.85 ± 11.18 years. Baseline population characteristics are listed in Table 2 (Ref. [2, 9, 10, 11, 12, 14, 20]). Four studies used Losartan [2, 9, 10, 11], two used Valsartan [12, 14] ,and one [20] used Candesartan.

**Table 1.** **Summary of the included studies**.

First author, year of publication	Country	Type of ARB	Dose of ARB	Control group	Follow-up	Measurement	Aim of the study	Conclusion
Kawano *et al*. 2005 [12]	Japan	Valsartan	80 mg/day	Conventional treatment without ARB	1 year	MRI	Effect of ARB on myocardial fibrosis in HCM.	Valsartan suppresses the synthesis of type I collagen in patients with HCM.
Yamazaki *et al*. 2007 [11]	Japan	Losartan	50 mg/day	Conventional treatment without ARB	1 year	MRI	Effect of ARB in the amelioration of myocardial impairment in HCM.	A single year of administration of ARB was sufficient to obtain a therapeutic effect on the natural course in patients with HNCM.
Penicka *et al*. 2009 [20]	Czech Republic	Candesartan	Initially 8 mg/day, doubled as tolerated every 2 weeks aiming for target dose of 32 mg/day	Placebo	1 year	TTE	Effect of long-term administration of ARB on LVH, left ventricular function, and exercise tolerance.	Candesartan induced regression of LVH, improved LV function, and exercise tolerance with no side effects in HCM.
Shimada *et al*. 2013 [2]	USA	Losartan	Initially 50 mg/day, increased to 100 mg/day if lower dosage was well tolerated after 1 week	Placebo	1 year	MRI	Effect of losartan on LVH and fibrosis in patients with HCM.	Losartan reduces the progression of myocardial hypertrophy and fibrosis by HCM.
Axelsson *et al*. 2015 [9]	Denmark	Losartan	Initially 50 mg/day, increased to 100 mg/day when initial dose was well tolerated after 14 days	Placebo	1 year	MRI, CT, or TTE	Effect of losartan on LVH and fibrosis in patients with HCM.	Losartan for 1 year did not reduce LVH compared with placebo in patients with overt HCM.
Axelsson *et al*. 2016 [10]	Denmark	Losartan	Initially 50 mg/day, increased to 100 mg/day when initial dose was well tolerated after 14 days	Placebo	1 year	MRI, CT, or TTE	If losartan could improve or ameliorate deterioration of cardiac function and exercise capacity.	Losartan had no effect on myocardial performance, disease progression, cardiac function, or exercise capacity compared with placebo.
Ho *et al*. 2021 [14]	4 countries	Valsartan	320 mg daily in adults; 80–160 mg daily in children	Placebo	2 years	ECG, CMR, CPET	To assess the safety and efficacy of valsartan in attenuating disease evolution in early HCM.	Valsartan improved remodeling in patients with early-stage HCM compared to placebo.

CMR, Cardiac Magnetic Resonance Imaging; CPET, Cardiopulmonary Exercise Testing; ECG, Electrocardiography; HNCM, hypertrophic nonobstructive cardiomyopathy; LVH, left ventricular hypertrophy; TTE, transthoracic echocardiogram; MRI, magnetic resonance imaging.

**Table 2.** **Baseline population characteristics**.

First author, year of publication	Total population	No. in the ARB group	No. in the control group	Age in the ARB group (mean ± SD)	Age in the control group (mean ± SD)	Female number (%)
Kawano *et al*. 2005 [12]	23	11	12	65 ± 7	62 ± 14	5 (21)
Yamazaki *et al*. 2007 [11]	19	9	10	55.4 ± 5.9	58.1 ± 8.8	0
Penicka *et al*. 2009 [20]	24	12	11	41 ± 15	45 ± 13	13 (54)
Shimada *et al*. 2013 [2]	20	11	9	49 ± 14	54 ± 11	3 (15)
Axelsson *et al*. 2015 [9]	133	64	69	51 ± 14	52 ± 12	47 (35)
Axelsson *et al*. 2016 [10]	133	64	69	51 ± 14	52 ± 12	47 (35)
Ho *et al*. 2021 [14]	178	88	90	23.1 ± 10.1	23.5 ± 10.1	69 (38)

ARB, angiotensin receptor blocker; SD, standard deviation.

### 3.3 Risk of Bias Assessment

**Fig. 2.**
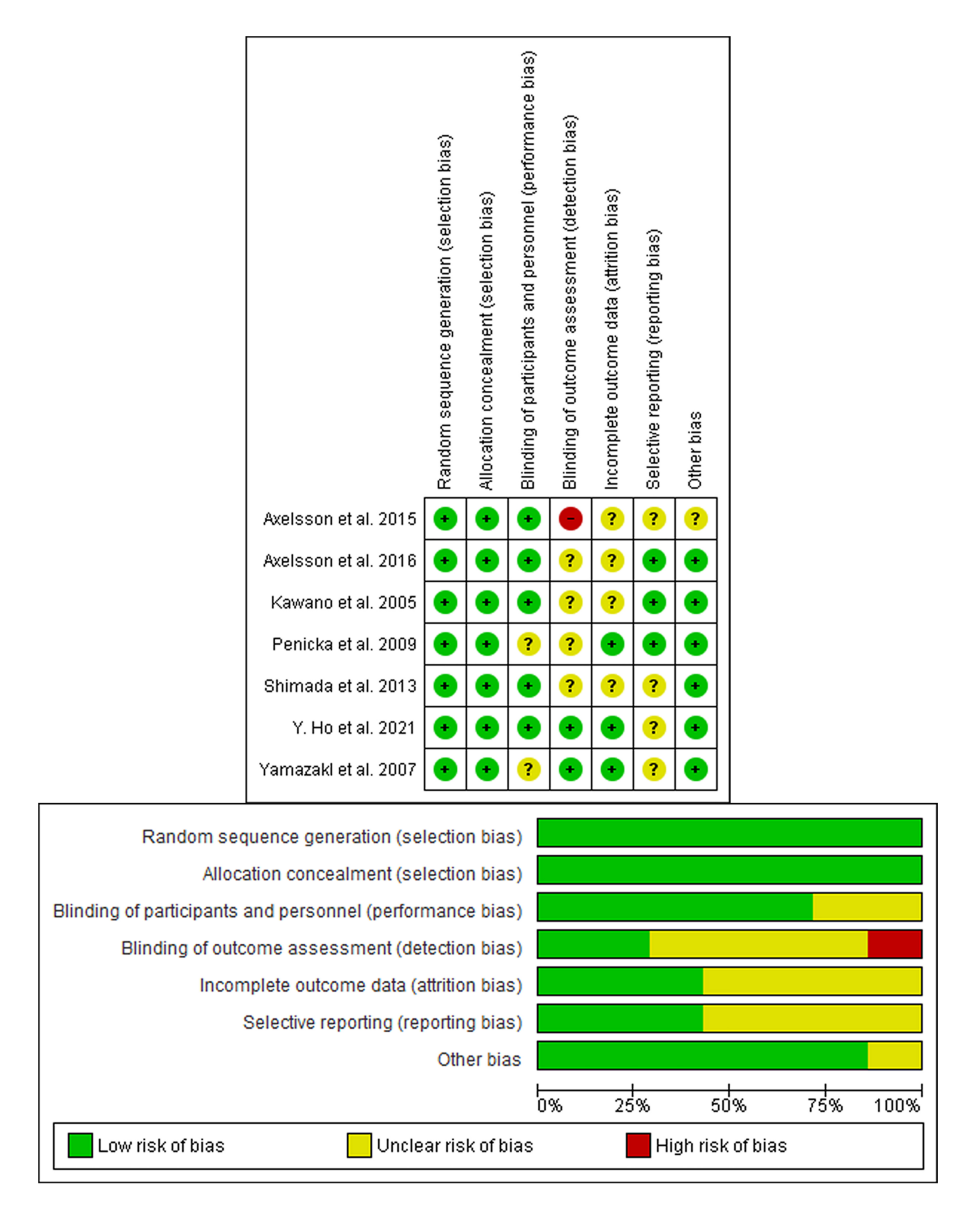
**Risk of bias assessment**. (A) Risk of bias graph: review authors’ judgments about each risk of bias item presented as percentages across all included studies. (B) Risk of bias summary: review authors’ judgments about each risk of bias item for each included study. The items are scored (+) low risk; (-) high risk; (?) unclear risk of bias.

Our results using ROB1 did not reveal any study with low quality; moreover, the summary of the results showed the high quality of the included randomized trials as represented in Fig. 2.

### 3.4 Outcomes 

#### 3.4.1 Primary Outcomes

**Fig. 3.**
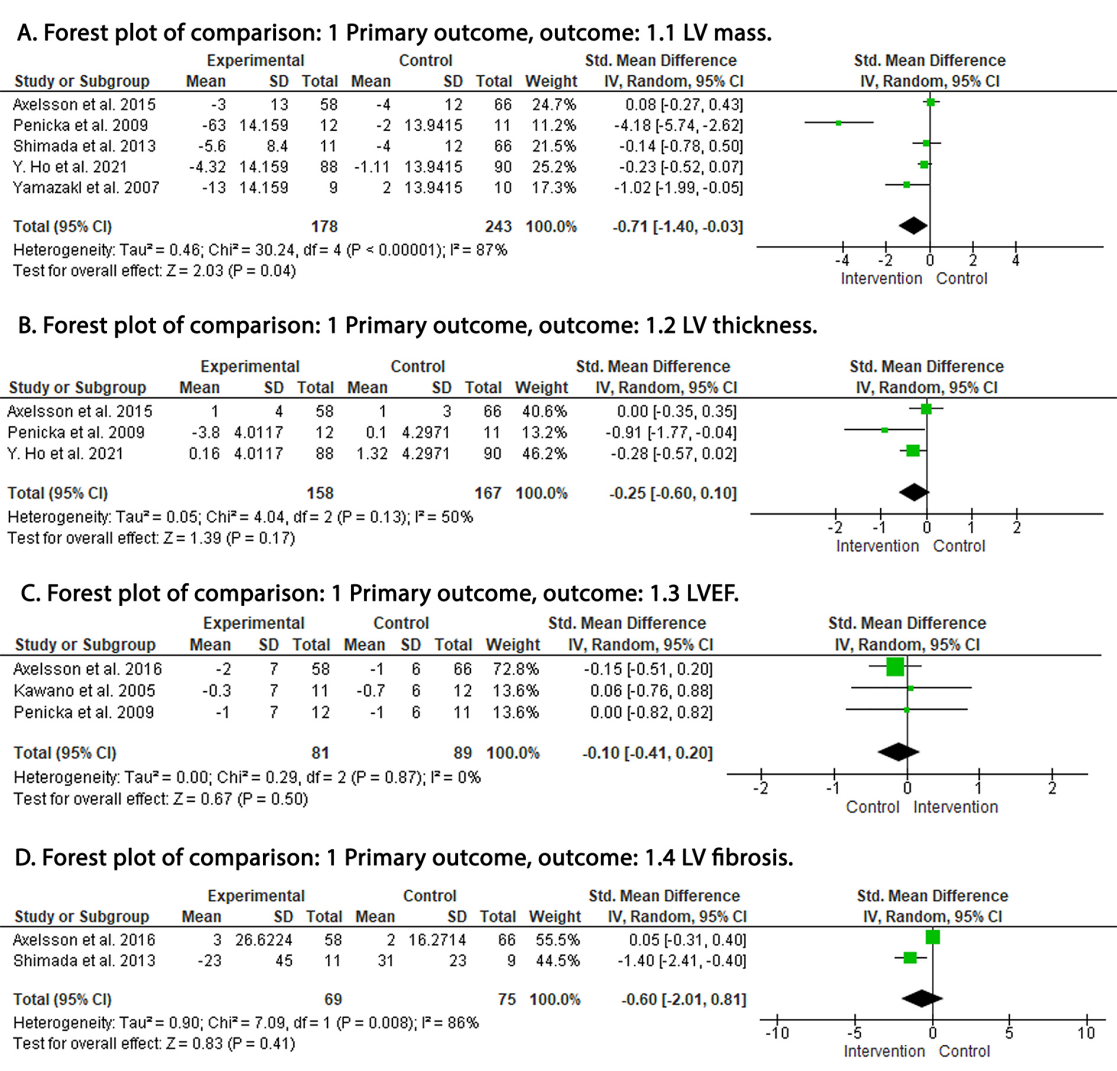
**Forest plot**. (A) LV mass. (B) LV thickness. (C) LVEF. (D) LV fibrosis. df, degrees of freedom; $I^{2}$, I-squared; IV, inverse variance; CI, confidence interval; LV, left ventricle; LVEF, left ventricular ejection fraction.

**Fig. 4.**
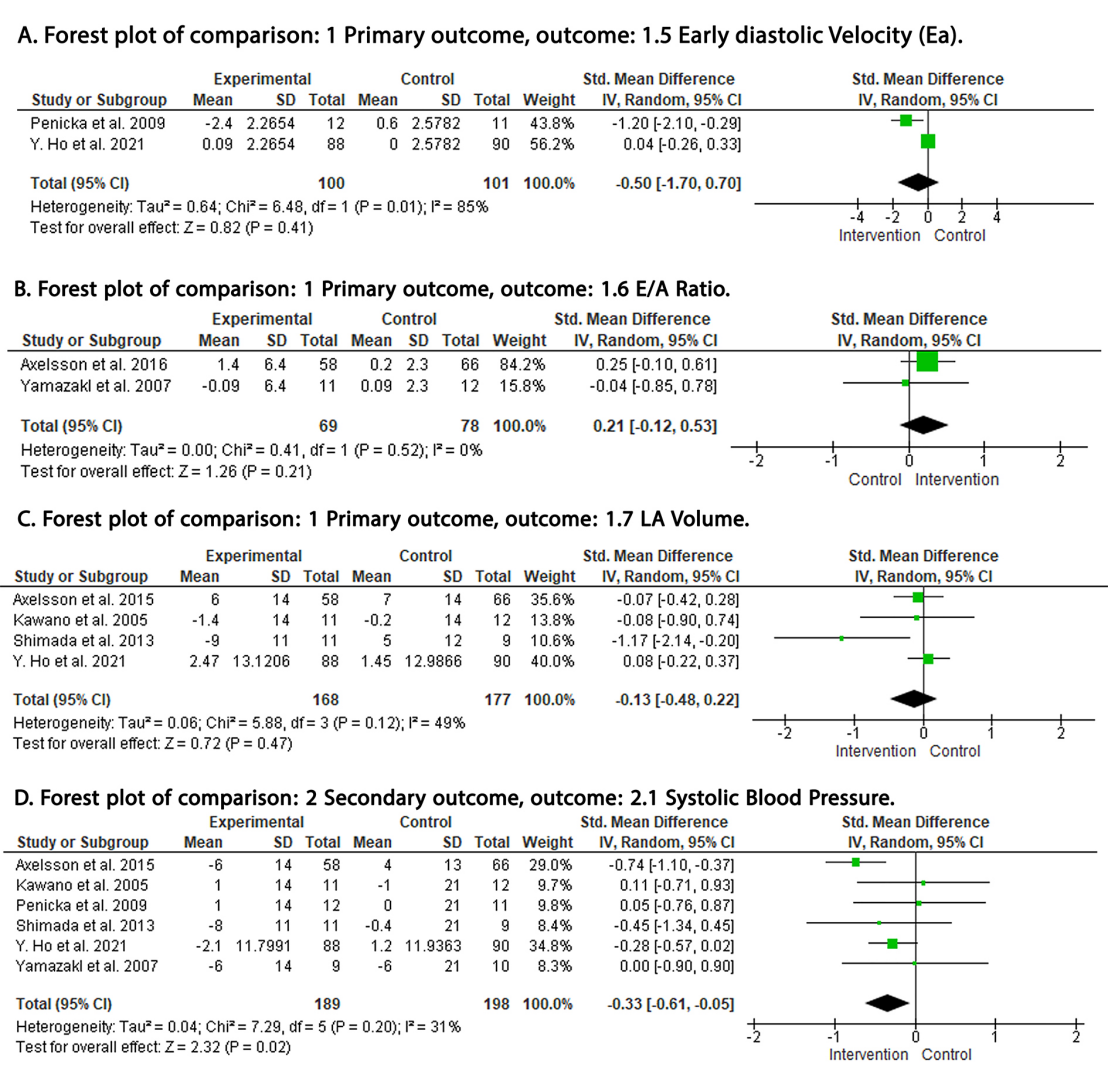
**Forest plot**. (A) Early diastolic velocity (Ea). (B) E/A ratio. (C) LA volume. (D) systolic pressure pressure. df, degrees of freedom; $I^{2}$, I-squared; IV, inverse variance; CI, confidence interval; LA, left atrial; E/A, early to late (atrial) transmittal flow velocities ratio.

LV mass was reported by five RCTs. Pooled analysis revealed that LV mass was significantly lower in the ARB group as compared to the control group (SMD: –0.77; 95% CI: –1.40, –0.03; *p* = 0.04; $I^{2}$ = 87%) (Fig. 3A). LV wall thickness was reported by three RCTs and there was no difference between ARB and control groups (SMD: –0.25; 95% CI: –0.60, 0.10; *p* = 0.17; $I^{2}$ = 50%) (Fig. 3B). LVEF was reported by three RCTs and was similar between ARB and control arms (SMD: –0.10; 95% CI: –0.41, 0.20: *p* = 0.50; $I^{2}$ = 0%) (Fig. 3C). LV fibrosis was reported by two RCTs with no significant difference between ARBs and control arms (SMD: –0.60; 95% CI: –2.01, 0.81; *p* = 0.41; $I^{2}$ = 86%) (Fig. 3D). Early diastolic velocity was reported by two RCTs and no significant difference was found between ARB and control groups (SMD: –0.50; 95% C: –1.70, 0.70; *p* = 0.41; $I^{2}$ = 85%) (Fig. 4A). Early to late (atrial) transmitral flow velocities (E/A) ratio was reported by two RCTs and there was no significant difference between ARB and control groups (SMD: 0.20; 95% CI: –0.12, 0.53; *p* = 0.21; $I^{2}$ = 0%) (Fig. 4B). Left atrial volume was reported by four RCTs and there was no significant difference between ARB and control groups (SMD: –0.13; 95% CI: –0.48, 0.22; *p* = 0.47; $I^{2}$ = 49%) (Fig. 4C).

#### 3.4.2 Sensitivity Analysis

Omitting the trial by Ho *et al*. [14] resulted in insignificant results (SMD: –1.07; 95% CI: –2.24, 0.09; *p* = 0.07; $I^{2}$ = 90%), also omitting Yamazakl *et al*. [11] or Penicka *et al*. [20] led to insignificant results. Detailed data about sensitivity analysis was represented in **Supplementary Table 4**, **Supplementary Material**.

#### 3.4.3 Subgroup Analysis

Subgroup analysis according to the type of used ARBs was not reliable due to the small number of available studies. However, our results showed significant results with the Candesartan subgroup (SMD: –4.18; 95% CI: –5.74, –2.62; *p ≤* 0.00001) (**Supplementary Fig. 1**, **Supplementary Material**).

### 3.5 Secondary Outcomes

Changes in systolic blood pressure were reported by six RCTs. Pooled analysis revealed significant blood pressure reduction in the ARB group (SMD: –0.33; 95% CI: –0.61, –0.05: *p* = 0.02; $I^{2}$ = 31%) (Fig. 2D). **Supplementary Table 5 **summarized the mean blood pressure in both the ARB group and the control group before and after the intervention.

## 4. Discussion

We conducted an updated systematic review and meta-analysis to compare the efficacy of ARB therapy in patients with HCM. Our results showed that ARB therapy was associated with a greater reduction in LV mass and systolic blood pressure as compared to the control group consisting of either placebo or standard non-ARB medication. There was no difference found in LA volume, LVEF, LV thickness, Ea, E/A ratio, and LV fibrosis between ARB and control groups.

The Role of renin-angiotensin system (RAS) inhibitors, including angiotensin-converting enzyme inhibitors (ACEi) and ARB, has been well documented in the prevention and potential reversal of myocardial remodeling secondary to hypertension [21, 22]. Conversely, aldosterone antagonists are another class of RAS inhibitors that have been implied to enhance cardiac remodeling and cause atrial fibrillation at higher dosages by increasing collagen synthesis and cardiac myocytes apoptosis [23]. Current European Society of Cardiology and American Heart Association guidelines for the management of HCM recommend initiation of RAS inhibitors in patients with LVEF <50% as part of guideline-directed medical therapy for heart failure (Class I recommendation, Level of evidence ‘C’). [7]. At present, ARB therapy is not mentioned as part of the routine medical management of patients with HCM in the absence of other indications such as reduced (<50%) LVEF [7]. Previously available data failed to show the efficacy of ARB therapy in patients with established HCM [2, 9, 10, 11, 12, 20]. Of note, many of these studies had several limitations, including smaller sample size and a shorter duration of follow-up (up to one year) [13].

Valsartan for Attenuating Disease Evolution in Early Sarcomeric Hypertrophic Cardiomyopathy (VANISH) trial began in April 2014 intending to test a novel strategy of disease modification in patients with sarcomeric HCM [14, 24]. The VANISH study showed improved HCM composite scores that incorporated overall cardiac structure and function [14, 24]. It is noteworthy that despite yielding a lower composite score for patients with sarcomeric HCM, individual reduction in LV mass and SBP were not significant in the ARB group of VANISH trial [14, 24]. In contrast, our pooled analysis of all RCTs did reveal a significant reduction in LV mass and SBP in the ARB group. This can be explained by the overall larger sample size and the addition of newer data from VANISH trial with early initiation of ARB and longer follow-up duration (two years). VANISH trial [14] had many fundamental differences in the study design as compared to other RCTs; (1) VANISH trial [14] included patients with confirmed sarcomeric HCM as compared to other trials who did not specify HCM etiology, (2) VANISH trial [14] included patients at a younger age (mean age 20–30 years versus 40–65 years in other RCTs), (3) VANISH trial [14] included patients with milder disease expression (LV wall thickness 16 mm versus 21 mm in other RCTs). It is also worth mentioning that despite being at higher risk for sudden cardiac death, most patients with HCM live a normal life with minimal to absent clinical manifestations [5, 25]. It is extremely challenging to prove the effectiveness of a treatment for such conditions with a wide spectrum of phenotypic manifestations and a relatively benign clinical course in most patients. VANISH trial [14] also showed that the most striking treatment benefits were seen in patients who were started on valsartan therapy in the early phase of HCM phenotypic expression.

It is historically reported in the literature that increased circulating angiotensin-II levels are associated with increased expression of TGF-β that in turn leads to interstitial fibrosis of various organs, including myocardium, vascular smooth muscle, liver, and kidneys [26, 27, 28, 29]. It is unknown at this time if a certain ARB agent or dosage is superior in decreasing TGF-β levels and halting myocardial hypertrophy and fibrosis. Our analysis includes just one RCT that used candesartan [20] in the ARB group, two RCTs [12, 14] used valsartan, whereas the remaining four RCTs [2, 9, 10, 11] opted to use losartan in HCM patients assigned to the ARB group. Amongst included studies, candesartan was administered at a dose ranging from 8–32 mg per day, valsartan dose ranged from 80–320 mg per day, and losartan was utilized in a dose range from 50 to 100 mg per day [2, 9, 10, 11, 12, 14, 20]. This difference in dose range was reported to be secondary to variability in patient tolerance and difference in study protocols.

Patients with HCM and evidence of left ventricular outflow tract (LVOT) obstruction are often treated with structural interventions including septal myomectomy or transcatheter alcohol ablation of septal hypertrophy (TASH) [30]. TASH is an alternative to septal myectomy and offers the same long and short-term mortality rate. However, compared to septal myectomy, TASH had a greater risk of right bundle branch block and applying permanent pacemakers and increased the demand for further septal reduction therapy [31].

Our study is an updated meta-analysis, including one additional study. First, our meta-analysis results are substantially different from the previous meta-analysis performed by Liu *et al*. [13] showing a significant reduction in LV mass in the ARB group. Secondly, the previous meta-analysis did not report systolic blood pressure, LV fibrosis, Ea, E/A ratio, and LA volume fibrosis as potential outcomes. Lastly, our analysis further emphasizes the importance of a larger sample size and longer follow-up duration for future trials studying the effectiveness of medical therapy for HCM.

There are a few potential limitations in our review. First, our study population was very heterogeneous, belonging to different age groups, and at different stages and severity of HCM phenotypes. Also, all included RCTs in our meta-analysis used MRI for the measurements of the endpoints, except Penicka *et al*. [20] used TTE. Despite echocardiography being a more feasible and affordable screening tool, magnetic resonance imaging provides more information and three-dimensional data and can diagnose the missed or query cases by ECHO [32]. Second, underlying genetic mutations were not specified by included studies except Ho *et al*. [14] that included only patients with sarcomeric HCM leading to the limited applicability of our data to HCM with specific genotypes. Third, the control groups were treated with standard medical therapy instead of placebo by two studies [11, 12] as compared to the other studies included in our analysis. Fourth, the included articles did not evaluate the circulating angiotensin II, catecholamines, or markers of oxidative stress and did not assess ACE nor angiotensin II type 1 receptor genetic polymorphisms. Those parameters could provide a deeper understanding of the effect of ARB in patients with HCM. Lastly, the longest follow-up duration was one year for most studies except Ho *et al*. [14] that reported two years of follow-up data leading to the limited applicability of our results over a longer follow-up period. We performed sensitivity analysis by removing Ho *et al*. [14] and Penicka *et al*. [20] as solutions to the above limitations, but the results were insignificant. Therefore, further research with a homogenous population is still needed.

## 5. Conclusions

In patients with HCM, ARBs are associated with significantly lower LV mass and a significant reduction in SBP as compared to non-ARB medication or placebo. Therefore, initiation of ARB therapy should be considered early in the disease course for patients with HCM. However, further RCTs using larger sample sizes and longer follow-up duration should be conducted to assess the validity and applicability of this study.

## Supplementary Material

Supplementary material associated with this article can be found, in the online version, at https://doi.org/10.31083/j.rcm2304141.
